# Beyond the Epidermal-Melanin-Unit: The Human Scalp Anagen Hair Bulb Is Home to Multiple Melanocyte Subpopulations of Variable Melanogenic Capacity

**DOI:** 10.3390/ijms241612809

**Published:** 2023-08-15

**Authors:** Cristina Casalou, Jay M. Mayatra, Desmond J. Tobin

**Affiliations:** 1 Charles Institute of Dermatology, School of Medicine, University College Dublin, D04 V1W8 Dublin, Ireland; 2 Conway Institute of Biomedical and Biomolecular Science, University College Dublin, D04 V1W8 Dublin, Ireland

**Keywords:** bulbar melanocytes, hair follicle (HF), follicular-melanin unit (FMU), melanin, C-KIT, MITF, SOX10, Melan-A

## Abstract

The visual appearance of humans is derived significantly from our skin and hair color. While melanin from epidermal melanocytes protects our skin from the damaging effects of ultraviolet radiation, the biological value of pigmentation in the hair follicle, particularly on the scalp, is less clear. In this study, we explore the heterogeneity of pigment cells in the human scalp anagen hair follicle bulb, a site conventionally viewed to be focused solely on pigment production for transfer to the hair shaft. Using c-KIT/CD117 microbeads, we isolated bulbar c-KIT-positive and c-KIT-negative melanocytes. While both subpopulations expressed MITF, only the c-KIT-positive fraction expressed SOX10. We further localized bulbar melanocyte subpopulations (expressing c-KIT, SOX10, MITF, and DCT) that exhibited distinct/variable expression of downstream differentiation-associated melanosome markers (e.g., gp100 and Melan-A). The localization of a second ‘immature’ SOX10 negative melanocyte population, which was c-KIT/MITF double-positive, was identified outside of the melanogenic zone in the most peripheral/proximal matrix. This study describes an approach to purifying human scalp anagen hair bulb melanocytes, allowing us to identify unexpected levels of melanocyte heterogeneity. The function of the more immature melanocytes in this part of the hair follicle remains to be elucidated. Could they be in-transit migratory cells ultimately destined to synthesize melanin, or could they contribute to the hair follicle in non-melanogenic ways?

## 1. Introduction

The hair follicle (HF), a mammal-specific mini-organ, plays a crucial role in skin homeostasis via its life-long cyclical tissue transformations characterized by distinct phases of active hair growth (anagen), partial involution (catagen), and relative rest (telogen) [1]. Such dramatic tissue remodeling impacts not only hair fiber production but more generally the skin vasculature, innervation, immune status, etc. [2,3]. During re-entry into a new anagen phase of the asynchronous human hair growth cycle, the HF ‘reconstructs’ its so-called follicular-melanin unit (FMU) to facilitate new pigmented hair shaft formation [4]. However, unlike epidermal melanocytes, the activity of some follicular melanocytes (e.g., melanogenically competent HF melanocytes) is tightly coupled to the hair growth cycle [2,5,6]. In this way, melanin produced by this highly differentiated and post-mitotic subpopulation of follicular melanocytes is transferred to proliferative and differentiating pre-cortical keratinocytes in the anagen hair bulb during the formation of the pigmented hair shaft. Although melanocyte subpopulations in the FMU and epidermal melanin unit (EMU) are derived from the same pluripotent cells of the neural crest during embryonic development, these pigment cell compartments remain distinct (but open) in adult human skin [7]. Another striking difference between the cell biology of the epidermis and HF pigmentation processes is the lack of post-biogenic change to melanin when transferred to cortical keratinocytes that make up the bulk of the hair fiber. In this way, the distal tip of often exceptionally long scalp fibers retains the same melaninization as the most recently formed proximal segments of the hair emerging from the scalp [4].

**Figure 1:**
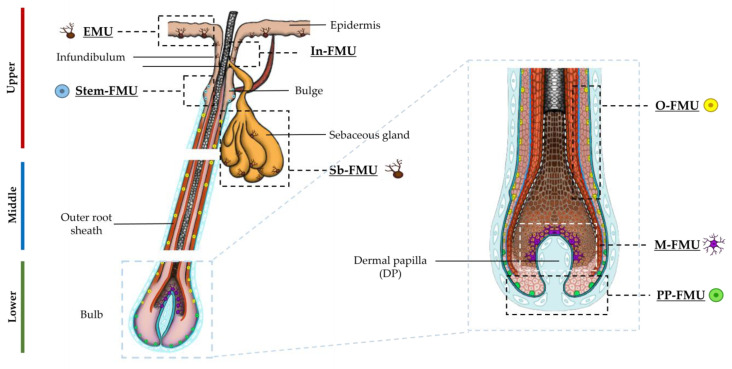
Schematic representation of human anagen HF, indicating the distinct melanin units (EMU, FMUs) present in the upper, mid, and lower regions of HF, and the distinct subpopulations of melanocytes of each melanin unit. Magnified schematics of the lower bulb region of HF is shown.

Follicular melanocytes during the hair-shaft-producing stage of the human hair growth cycle (i.e., Anagen VI) are extraordinarily diverse [4]. At this stage of the hair cycle, where the HF is at its maximum tissue size (up to 4 mm deep in scalp skin), follicular melanocytes are largely sub-defined by their tightly restricted anatomic location (Figure 1), size, morphology, and maturation/differentiation status. The latter is the most easily recognized melanogenic activity (amelanotic versus eu/pheomelanic) [2,8]. The tight coupling of melanogenesis to the anagen phase of the hair growth cycle facilitates very significant remodeling of follicular melanocyte subpopulations when full anagen HF transits into telogen. Only melanoblasts and some catagen-surviving immature melanocytes can be readily detected during this period of relative rest. Still, they can be recognized as typically non-dendritic, small cells with oval nuclei and clear cytoplasm that lack readily detectable (pre)melanosomes. Importantly, they lack the features of epithelial cells (e.g., desmosomal junctions) or Langerhans cells (indented nuclei, Langerhans granules). During the first three to four decades of human life (i.e., before the onset of hair graying), most pilosebaceous units in the adult scalp contain a phenomenally active group of melanocytes, in terms of their melanin synthesis, located in the anagen hair bulb (M-FMU) [9,10].

There, a relatively small number of post-mitotic and terminally differentiated melanocytes crosstalk with neighboring keratinocytes and dermal papilla fibroblasts to produce extraordinary amounts of melanin, year after year, to pigment scalp hair fibers that can be over a meter long. In this way, these M-FMU melanocytes experience a very different reality to similarly melanogenically active melanocytes in the HF infundibulum (In-FMU) or basal layer of the sebaceous gland (Sb-FMU), likely due to very different relationships with their partner keratinocytes and adjacent mesenchyme [8]. 

At the other end of the pigmentary spectrum resides a diversity of amelanotic or poorly differentiated melanocytes and/or melanoblasts. The latter cells reside in the upper hair follicle close to the insertion site of the arrector pili muscle (euphemistically referred to as the HF ‘bulge’ region, although human HFs (unlike those in mice) do not have an anatomically distinct bulge). This region contains HF melanocyte stem cells (Stem-FMU). Amelanotic melanocytes are also located 1–3 mm more proximally in the skin in the mid-to-lower outer root sheath (O-FMU), as well as in the most peripheral proximal bulb region (PP-FMU) of the growing anagen HF (Figure 1) [2,8]. The nature of the relationships between the three ‘immature’ melanocyte subpopulations (i.e., Stem-FMU, O-FMU, PP-FMU), between the four ‘mature’ melanocytes subpopulations (i.e., EMU, In-FMU, Sb-FMU, and M-FMU), and between both ‘mature’ and ‘immature’ pigment cell populations remains enigmatic. For example, it is not yet clear whether at least some of these pigment cell subpopulations can move or migrate throughout the HF during the anagen hair fiber growth phase of the hair growth cycle.

Much of our knowledge of melanocyte development and subpopulation heterogeneity has been obtained and deduced from inbred laboratory mice. These studies have been crucial for discovering genes underlying hair cycle control and general pigmentation. Inevitably, however, the distinct hair growth cycle (mouse synchronous versus human mosaic) and other cell biological and enzymological features of HFs between the nocturnal mouse and diurnal human, particularly around the regulation of skin and HF pigmentation, limit the accurate translation of findings from mouse models to human. Still, markers of murine HF melanocyte lineages have provided at least a starting point for the exploration and identification of melanocyte plasticity in human skin and include markers for melanogenesis/melanin synthesis, (e.g., MITF, DCT/Trp2, Trp1) and melanocyte development, differentiation, and survival (e.g., SOX10, c-KIT, MITF), among others [2,6]. While much of current cutaneous pigmentation research continues to reside in the mouse domain, the identity and characterization of heterogeneous subpopulations of melanocytes of human scalp HFs is a fertile area of research where many important questions in human HF pigmentation science have yet to be addressed.

This study addresses whether melanocytes in the growing human (anagen) scalp HF bulb exist along a differentiation/maturation spectrum, characterized here by the expression of so-called ‘early’ and ‘late’ maturity markers in vivo. Using the receptor tyrosine kinase cell surface marker protein c-KIT (CD117) that identifies ‘precursory’ melanocytes in human skin [11,12], we isolated melanocyte subpopulations from three distinct follicular units from the lower portion of human anagen HFs i.e., O-, PP-, and M-FMUs (Figure 1). These reflect melanocytes with variable levels of cell differentiation. The SCF/KIT pathway plays a critical role in the control of normal human melanocyte homeostasis [12], and c-KIT expression can demarcate a population of precursor melanocytes in human HF.

## 2. Results

### 2.1. Distinct Melanocyte Subpopulations Can Be Isolated in Culture from the Adult Human Anagen Scalp Hair Bulb along a Differentiation/Maturation Spectrum

**Figure 2:**
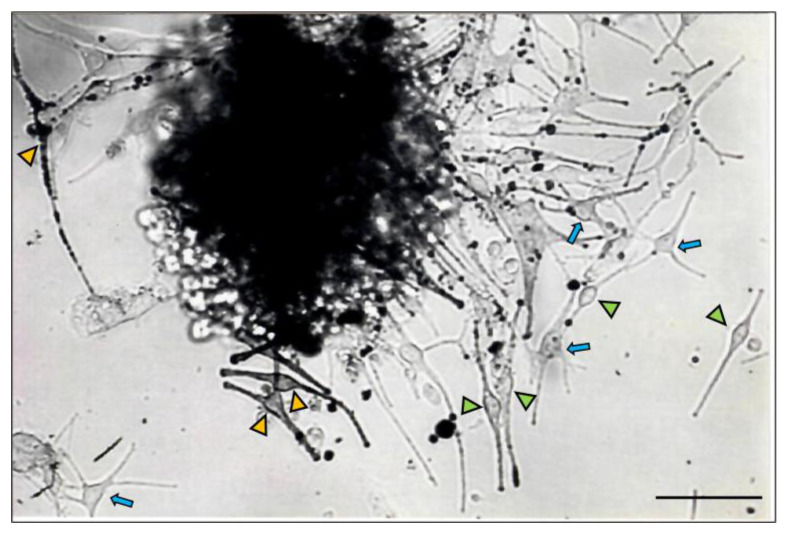
Primary bulbar melanocytes of adult human scalp HF in primary culture (day 7), showing the presence of bipolar HF melanocytes (arrowheads), some intensely pigmented (yellow arrowheads), others fainter with low in melanin content (green arrowheads), together with poly-dendritic melanocytes with variable pigment content (blue arrows). Bar = 20 μm.

The proximal hair bulb region is the main epithelial (keratinocyte) proliferative region of the growing (anagen) HF and has generally been viewed as harboring the differentiated and melanogenically active melanocyte population (M-FMU). Using a histoculture approach, we isolated from the HF bulb a pool of melanocytes (Figure 2) with recognizable and highly variable morphologies (poly-dendritic, bipolar vs. fusiform, etc.).

**Figure 3:**
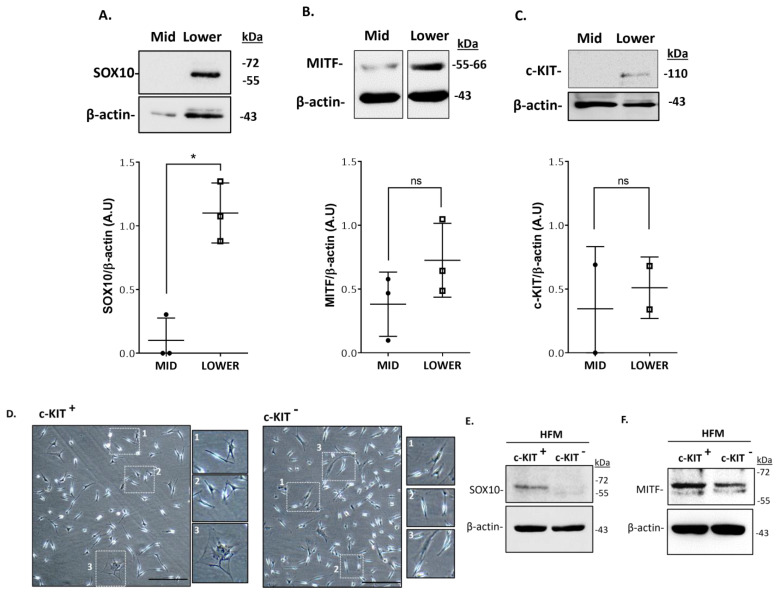
Isolation of melanocytes from adult human scalp HFs restricted to the mid-lower bulb region. Total protein extracts prepared from microdissected human follicles sectioned in mid and lower regions were analyzed by Western blot analysis for the expression of SOX10 (**A**), MITF (**B**), and c-KIT (**C**). Relative quantification of band intensities was made using β-actin or α-tubulin as loading controls. Data are the mean ± SD * *p*-value< 0.05; ns indicates not significant (non-parametric t-test; Wilcoxon). Further enrichment of melanocyte populations was made using CD117/c-KIT selection (MACS technology). (**D**) Phase contrast images representing the morphology of CD117-positive and CD117-negative cells at passage 1 (P1); attached bipolar and polydendritic cells (enlargements 1,2) and stromal cell (incl. fibroblast) contamination (enlargement 3). Scale bar 50 μm. SOX10 expression (**E**) was confirmed only in CD117-positive cell fraction, by Western blot analysis whereas MITF expression (**F**) was detected in both cell populations, (c-KIT^+^; c-KIT^−^). β-actin was used as a loading control.

Additionally, these cells displayed distinct pigmentation levels (amelanotic, low pigmented, and highly pigmented), suggesting that the anagen hair bulb contains heterogeneous populations of melanocytes with distinct differentiation stages and melanogenic activity. However, the function of these cells beyond the capacity for melanin synthesis per se is unclear. To characterize the populations of melanocytes of the mid-lower region of human HFs (Figure 1), we start by extracting the total protein content of microdissected human anagen VI HFs (see Table S1 for HF donors’ details). After the separation of the mid and lower regions of the follicles, we analyze by Western blotting the expression of markers of melanocyte development and differentiation in these two distinct fractions of HFs (i.e., SOX10, MITF, and c-KIT) (Figure 3; Supplementary Figure S1). Whereas SOX10 expression was detected only in the lower bulb region of HFs, MITF and c-KIT were expressed in both regions (mid and lower) of HFs (Figure 3; Supplementary Figure S1). Due to the relatively low number of melanocytes present in each adult scalp HF (relative to keratinocyte and fibroblast numbers) and their limited ability to proliferate in culture, the unbiased culture of all bulbar melanocyte subpopulations can be very challenging. After the initial steps of the isolation of melanocytes from human HF scalp tissue, modified from the original methods reported by Tobin and co-workers [13,14], we selected melanocytes based on their relative expression of the cell surface marker CD117/c-KIT. This allowed us to enrich HF melanocytes from the mid to lower HF regions using MACS technology (Figure 3D–F) [15]. C-KIT is expressed in both melanocytes and several stem cell populations throughout the human body [12,16]. Binding to its ligand, stem cell factor (SCF) plays an important role in cell homeostasis, including in cell survival, migration, and proliferation [12,17]. We observed the isolation and expansion of melanocytes in both CD117/c-KIT^+^ and CD117/c-KIT^−^ melanocyte fractions along with apparently some stromal/fibroblast-like cell growth (Figure 3D). A Western blot analysis confirmed the expression of the transcription factor SOX10 in the c-KIT^+^ melanocyte population (Figure 3E), whereas the transcription factor MITF (Figure 3F) was expressed in both c-KIT^+^ and c-KIT^−^ cell populations.

This suggests the existence of at least two distinct subpopulations of melanocytes in the proximal bulb region of human HFs, expressing markers of melanocyte development and differentiation (i.e., SOX10, c-KIT, and MITF).

### 2.2. Localization and (Co)-Expression of Melanocyte Differentiation Markers in the Human Anagen Scalp Hair Bulb

**Figure 4:**
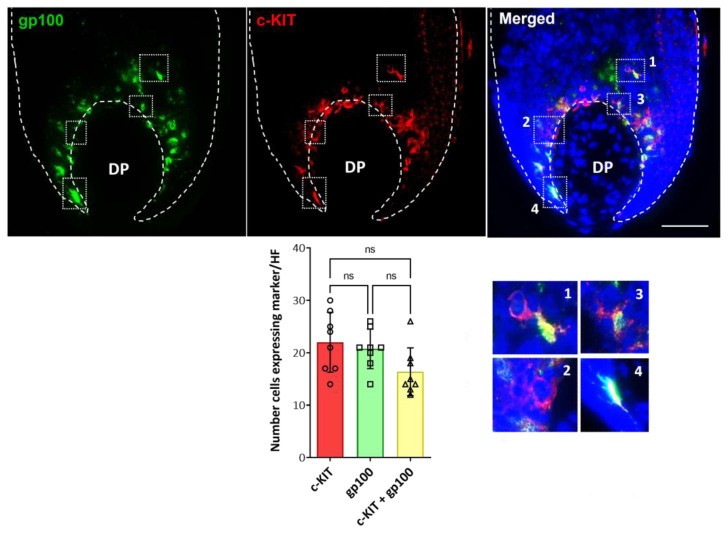
In situ localization of c-KIT expression in the proliferative bulb region of human adult scalp HF. c-KIT positive melanocytes were detected that lacked the differentiation marker, the (pre)-melanosomal protein gp100 (enlargements #1,2), while other c-KIT-positive melanocytes were already expressing gp100 (enlargements #3,4) (n = 3). Cell nuclei are indicated by DAPI staining. DP—dermal papilla. Scale bar 50 μm. The plot represents the quantification of the number of cells positive for each marker (gp100, green; c-KIT, red), and double-positive cells (n = 8; 4 donors). Data are the mean ± SD; ns indicates not significant (ordinary one-way ANOVA test).

To study the distribution of distinct populations of follicular melanocytes present in the bulb region of human growing HFs, cryosections of occipital scalp samples were subjected to immunohistochemical analysis (i.e., antibodies to c-KIT, SOX10, MITF, gp100, Melan-A; Table S2). In the bulb region of HFs around the Auber’s line level of DP, where melanin is actively transferred from mature melanocytes to pre-cortical keratinocytes that color the hair fiber, we identified differentiated melanocytes using the (pre)-melanosome marker gp100 (Figure 4; Supplementary Figure S2). However, c-KIT-positive melanocytes not expressing gp100 were also detected in the same region of the hair bulb (Figure 4; enlargements #1–2 compared with #3–4). Despite this, no significant differences were found in the number of cells that expressed each marker and the double-positive cells (Plot of Figure 4; Supplementary Figure S2).

**Figure 5:**
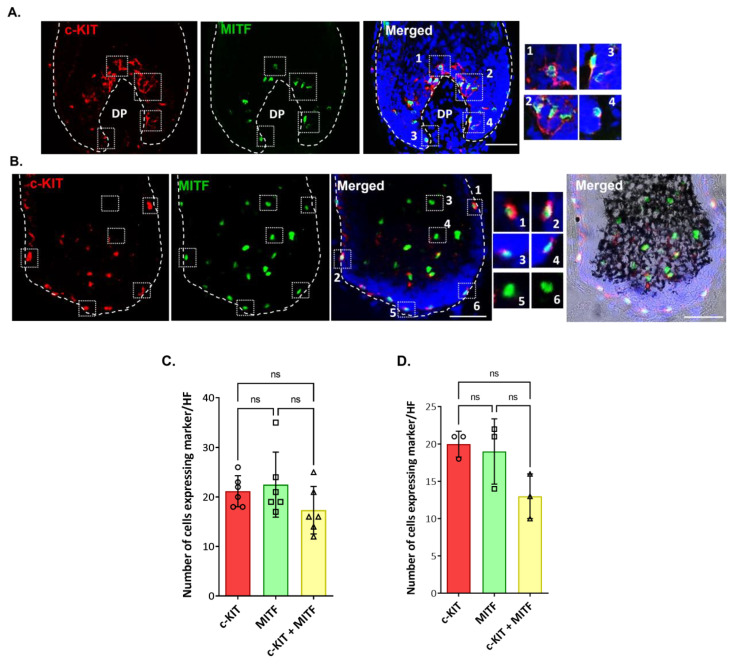
c-KIT is variably co-expressed with MITF by melanocytes of the PP-FMU and M-FMU. Longitudinal cryosections of human adult scalp HFs were taken from the interior (**A**) and the exterior (**B**) regions and used to examine the expression of c-KIT and MITF (n = 2). (**A**) Magnifications 1–4 highlight the cellular co-localization of nuclear MITF with the cell membrane/cytoplasmatic c-KIT expression in the same cells of the melanogenically active region of HFs. (**B**) Melanocytes present at the peripheral proximal region (PP-FMU; enlargements #1–4) co-express c-KIT and MITF, whereas in the highly proliferative region of the hair bulb matrix (M-FMU) some melanocytes can be found that are only positive for MITF (enlargements #5–6). Cell nuclei are indicated by DAPI staining. Scale bar 50 μm. DP—dermal papilla. BF—Brightfield. (**C**,**D**) Quantification of the number of cells positive for each marker (c-KIT, red; MITF, green), and double-positive cells (n = 6; 3 donors in (**A**), n = 3, 3 donors in (**B**)). Data are the mean ± SD; ns indicates not significant (ordinary one-way ANOVA test).

We also detected c-KIT expression (plasma membrane/cytosol) in MITF-positive (nuclear expression) and melanogenically active melanocytes present above the DP (Figure 5A; enlargements #1,2; Supplementary Figure S3). However, c-KIT and MITF co-expression was also detected in melanocytes of the peripheral proximal region (PP-FMU) (Figure 5B, enlargements #1–4), i.e., outside the melanogenic zone of the hair bulb. To examine hair bulb melanocytes that are distributed at some distance from the DP-hair matrix interface, we took longitudinal cryosections of HFs that did not transect the DP. Here, we unexpectedly observed that some MITF-positive melanocytes were c-KIT-negative (Figure 5B; enlargements #5–6) or with low expression detectable levels.

**Figure 6:**
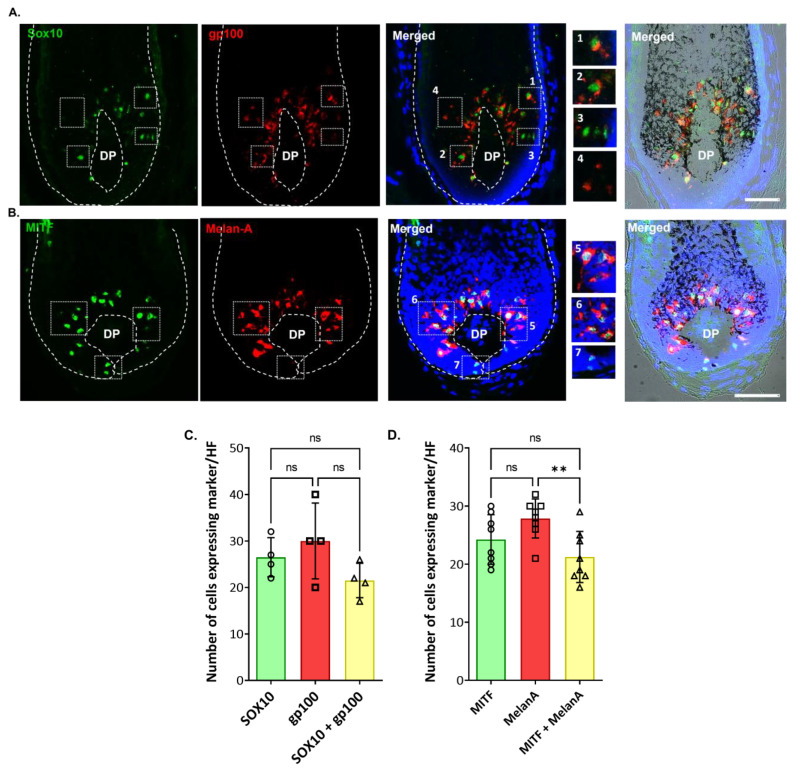
Melanocyte expression of transcription factors MITF and SOX10 (in the proliferative bulb region of Human anagen HFs. The expression of SOX10 and MITF was assessed by IHC (**A**), together with melanocyte/melanosome markers gp100 and Melan-A (**B**). Enlargements #1–4 highlight the cellular localization of SOX10 and gp100, whereas #5–7 highlight the cellular localization of MITF and MelanA. Some melanocytes localized above the DP were SOX10 positive, but not yet making significant melanin (i.e., lacked gp100 expression; see enlargement #3), and others did not express SOX10 (enlargement #4). Cell nuclei are indicated by DAPI staining. DP—dermal papilla. Scale bar 50 μm. (**C**) Quantification of the number of cells positive for each marker (SOX10, green; gp100, red) and double-positive cells (n = 4; 3 donors). (**D**) Quantification of the number of cells positive for each marker (MITF, green; MelanA, red) and double-positive cells (n = 8; 3 donors). Data are the mean ± SD ** *p*-value < 0.01 and ns indicates not significant (ordinary one-way ANOVA test).

Furthermore, in the proximal HF bulb region, we identified melanocytes expressing the melanocyte marker of differentiation SOX10 (Figure 6; Supplementary Figures S4 and S5).

Interestingly, some melanocytes exhibited the co-expression of both SOX10 and gp100 (Figure 6A,C, enlargement #1,2), suggestive of melanocyte differentiation (i.e., capacity to make melanosomes and synthesize melanin), while other SOX10-positive melanocytes appeared to be immature (non-melanic), as assessed by the absence of co-expression for the melanosome marker gp100 (Figure 6A; enlargement #3). Similarly, distinct sub-populations of MITF-positive melanocytes in the PP-FMU were positive for the melanocyte differentiation antigen Melan-A (Figure 6B,D, enlargement #5,6), while other MITF-positive melanocytes in the PP-FMU lacked Melan-A (Figure 6B, enlargement #7). No significant differences were observed in the expression of both SOX10 and MITF (Figure 6C,D; Supplementary Figures S4 and S5). This suggests the presence of melanocytes in different differentiated states in the M-FMU. In summary, distinct hair bulb melanocyte populations can be distinguished by the expression of pigment cell differentiation markers SOX10 and c-KIT in the melanogenically active bulb region of HF, together with the expression of the master pigment cell transcription factor MITF and down-stream differentiation-associated melanosome markers gp100 and Melan-A.

### 2.3. Dopachrome Tautomerase (DCT/TRP-2) Is Expressed by Melanocytes in the Human Scalp Hair Bulb, which Can Be Upregulated Further by Inflammatory (IFN-γ) and UVR Stressors

**Figure 7:**
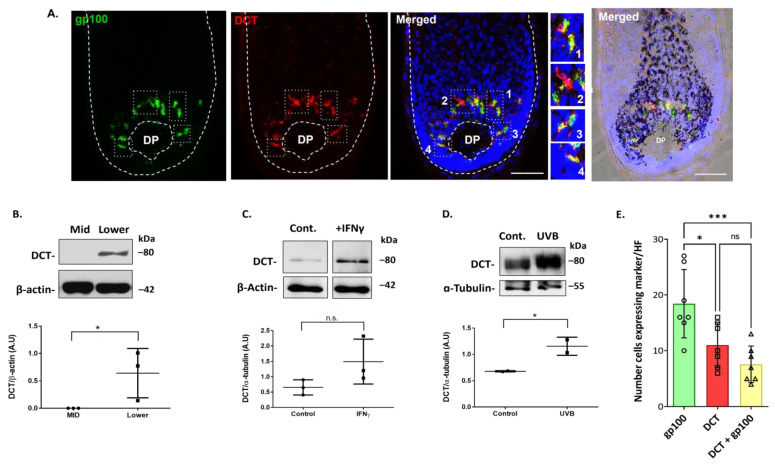
DCT was expressed by melanocytes in the melanogenically-active zone of the adult human scalp anagen bulb. (**A**) IHC analysis of DCT expression in the HF bulb region, where some melanocytes (red-only) expressed DCT, but expressed little or no gp100+ melanosomes (green). Enlargements #1–4 highlight DCT and gp100 cellular localization. DAPI -nuclear staining; DP—Dermal papilla. Scale bar 50 μm. (**B**) DCT was expressed in protein extracts of the melanogenic hair bulb, but not in protein extraction from the mid-region of HF, where O-FMU melanocytes reside (n = 3, 3 donors). IFNγ treatment (**C**) and UVB-induced stress (**D**) both upregulated DCT protein expression in the lower anagen hair bulb fractions, (n = 3, n = 2 respectively). β-actin and α-tubulin were used as loading controls. * *p*-value < 0.05 and ns indicates not significant (non-parametric *t*-test; Wilcoxon). (**E**) Quantification of the number of cells positive for each marker (DCT, green; gp100, red) and double-positive cells (n = 4; 3 donors). Data are the mean ± SD *** *p*-value < 0.001 * *p*-value < 0.05 and n.s. indicates not significant (ordinary one-way ANOVA test).

It has been reported that DCT/Trp2 is expressed in immature melanoblasts of the human HFs (Stem-FMU) [18]. Additionally, DCT is expressed by most melanoma cells, and it has been used as a melanoma differentiation antigen in diagnostic histopathology [19]. However, it was previously reported that melanogenically active melanocytes may lack the expression of DCT protein, at least in human scalp HF [20]. Still, given the prominence of DCT in the melanogenesis enzymatic pathway [21], we decided to re-examine DCT expression in HF melanocyte subpopulations in the normal human scalp. Using two distinct and validated commercial antibodies to DCT (Table S2), we localized DCT expression in the melanogenically active bulb region of adult human scalp HFs (Figure 7A; Supplementary Figure S6). 

DCT/Trp2 expression was detected in both fully differentiated melanocytes of the bulb region and in melanocytes with low or absent melanosome biogenesis, as evidenced by weak/no expression of gp100 (Figure 7A, enlargement #2). Of note, the number of cells expressing DCT was significantly reduced relative to the number of gp100-positive cells (Figure 7E; Supplementary Figure S6). DCT expression in this melanogenically restricted region of human scalp HFs was further confirmed by a Western blot analysis of the total protein content of the proximal bulb region of HF (lower) (Figure 7B; Supplementary Figure S6B). By contrast, DCT expression was not detected in protein extracts derived from the mid-region of HF immediately distal to the melanogenically active bulb (Figure 7B) at the level that contained some amelanotic outer rot sheath melanocytes (O-FMU). 

The ex vivo organ culture of isolated HFs from the human scalp that was exposed to specific stress mediators (i.e., IFNγ treatment and UVB irradiation) exhibited the induction of DCT protein expression over unstimulated controls (Figure 7C,D; Supplementary Figure S6).

## 3. Discussion

The trajectories encountered by pigment cells in mammalian skin from their first commitment in the neural crest during development [22] to their proliferation and differentiation as fully-committed cutaneous melanocytes of the skin and hair follicle [23], ultimately to their demise episodically during hair follicle regression (catagen) or essentially permanently during canities (hair graying) has been best described in mice [24], though some data for humans has begun to emerge [25,26]. We recently reviewed the multiplicity of melanocytes in the adult human scalp hair follicle [8] and in the present study re-examined whether some of this multiplicity is also extended to the anagen hair bulb, which we conventionally see predominantly as the melanogenic center of the growing HF [4].

The initiation of HF melanocyte primary cultures from human scalp follicular tissue, limited to the anagen hair bulb region, revealed the explanation of a remarkable diversity of pigment cell morphologies (Figure 2). The characteristic appearance of these explants before the proliferation of some cells occurred attests to the pre-existence of a range of pigment cell maturity states that are both tissue-restricted and, importantly, only transient, i.e., are dramatically remodeled during the hair growth cycle that sees the loss of the anagen hair bulb during catagen [27].

One of the challenges of this field of melanocyte heterogeneity in adult human skin and HF is the scarcity of non-pigment cell-differentiation-associated markers and associated reagents that can detect immature melanocytes. Specifically, amelanotic and non-dendritic melanocytes/melanoblasts are difficult to detect, given the lack of specific markers, whereas the fully differentiated melanocytes of the bulb region (M-FMU) have been identified in HFs by their expression of melanocytic markers such as MITF, tyrosinase and tyrosinase-related proteins 1 and 2 (TRP1 and DCT/TRP2, respectively) [2,6]. Additionally, human HF melanocytes have been identified, isolated, and cultured [13,14]. However, it is our view that by using these methods (Figure 3), we can selectively expand the immature melanocytes of the ORS (O-FMU). Thus, still more robust methods are needed to isolate and propagate HF melanocytes that are representative of all subpopulations in HF, including hair bulbs in vitro, to better functionally characterize these cells. Recently, c-KIT was used to isolate and enrich melanocyte populations derived from human corneal limbus [28] and melanocyte precursor cells present in the human limbal stroma [29]. C-KIT, a receptor tyrosine kinase protein, and its ligand stem cell factor (SCF) are required for the cyclic regeneration of the hair pigmentary unit and migration of melanoblasts in developing murine hair follicles [12,30,31]. In this study, we successfully isolated and expanded c-KIT-positive and c-KIT-negative populations of bulbar melanocytes of relatively similar morphologies. The melanocytic identity of the latter c-KIT-negative subpopulation was confirmed by the expression of MITF, while only the c-KIT-positive subpopulation additionally expressed readily detectable levels of the transcription factor SOX10.

We have previously shown in developing mouse HFs that melanoblasts express c-KIT as a prerequisite for migration into the SCF-supplying HF epithelium [32]. Importantly, differentiated c-KIT-positive melanocytes target the HF bulb, while c-KIT-negative melanoblasts invade both the ORS and bulge in the fully developed HF. Thus, we confirm here for the human hair bulb, the presence of melanocytes expressing markers of early pigment cell differentiation, such as c-KIT and additionally SOX10, together with melanocytes positive for more mature markers like MITF, gp100, and Melan-A. SOX10 as a neural crest marker can be considered an early differentiation melanocyte marker [33] and in melanoma cells has been associated with melanoma cell proliferation, tumor formation, and growth [34,35]. SOX10 has been shown to heavily influence melanocyte development in adults, while SOX9 has been implicated in melanogenesis [36]. This is supported by our finding that some SOX10-positive melanocytes in the anagen hair bulb appeared to be relatively immature (non-melanic), as evidenced by the absence of the melanosome marker gp100.

Importantly, the variable expression of c-KIT in hair bulb melanocytes indicates that some pigment cells are more mature than others. Specifically, the sole expression of c-KIT (i.e., without additional expression of gp100) in melanocytes suggested that these cells are not (yet) competent for melanosome biogenesis and subsequent melanogenesis. In this way, these melanocytes are positioned differently on the spectrum of pigment cell differentiation to closely located melanocytes that exhibit a co-expression of c-KIT with gp100. No other cell types resident within (or even transient visitors to) the human anagen hair bulb matrix have been reported to express c-KIT (mast cells are not found in the hair bulb matrix epithelium). It is important to demarcate the hair bulb matrix epithelium from the connective tissue areas of the anagen hair bulb (including dermal papilla and dermal sheath components), as the latter are vascularized tissues and can readily accommodate in-transit cells of non-fibroblast and non-endothelial lineages (e.g., mast cells). There have been recent studies, albeit in altogether different tissue types, reporting the isolation of melanocyte populations from the human limbal stroma [28,29] using c-KIT antibodies. It is important to note that corneal stromal stem cells are a population of neural-crest-derived mesenchymal (stem) cells residing outside the self-renewing corneal epithelium. Data (partly) supporting our observations in the current study, albeit not from hair follicles, include reports of non-pigmented c-KIT-positive melanocyte precursors in the human interfollicular epidermis [37]. In this study, three populations of melanocytes were identified in the human epidermis, two of which expressed c-KIT, while the third population was described as cKIT-CD90+. Those authors concluded c-KIT to be a marker restricted to melanin-producing melanocytes. However, this was clearly not the case in the human anagen hair bulb, where c-KIT expression was present in the absence of gp100 expression—a marker of (pre)melanosome biogenesis. This suggests the existence of at least two distinct subpopulations of melanocytes in the proximal bulb of human HFs, characterized by the differential expression of markers of melanocyte development and differentiation (i.e., SOX10, c-KIT, and MITF). Support for this interpretation can be found in other expression pathways in the hair follicle pigmentary unit, including for hypothalamus-pituitary-adrenal axis components (e.g., α-MSH, ACTH, or CRF and their respective receptors), which are expressed in melanocytes of the PP-FMU but not the M-FMU [38].

Melanocyte differentiation is largely dependent on the expression of SOX10 and MITF, the master regulator of melanocyte development, function, and survival. The best-characterized transcriptional target of SOX10 in melanocytes appears to be MITF, and numerous studies demonstrated that SOX10 directly activates MIFT transcription [39]. The expression of SOX10 in melanocytes localized in the melanogenic zone above the DP of adult scalp human HFs and in the c-KIT positive fraction (but not/much less in the c-KIT-negative fraction) of isolated hair bulb melanocytes indicates the presence of melanocytes of distinct differentiation stages in this region. We suggest that c-KIT expression per se does not itself confer permissiveness or capacity for melanogenic potential in hair bulb melanocytes, as c-KIT-positive melanocytes may or may not lack co-expression of the (pre)-melanosomal marker gp100, though all c-KIT-positive melanocytes expressed MITF. By contrast, the corollary does not appear to be true i.e., while all hair bulb melanocytes are defined by their intrinsic MITF expression, not all MITF-positive melanocytes additionally express c-KIT, Melan-A, or gp100. Thus, distinct hair bulb melanocyte populations can be distinguished by the expression of the so-called ‘early’ pigment cell differentiation markers SOX10 and c-KIT in the melanogenically active bulb region of HF, together with the expression of the master pigment cell transcription factor MITF, and down-stream differentiation-associated melanosome markers gp100 and Melan-A.

Despite the key role of DCT/TRP2 in melanin biosynthesis, this protein is also one of the markers of the earliest melanocyte differentiation reported in mouse HF [18,40]. Indeed, Wegner et al. first reported that melanocyte-specific expression of DCT, a direct target of SOX10, was dependent on synergistic gene activation by both SOX10 and MITF transcription factors in mouse HF melanocytes [41]. Moreover, MITF synergistically enhances the SOX10-dependent activation of the DCT promoter. Thus, it appears anomalous that DCT expression in the melanogenic bulb HF region would be lacking for human scalp anagen hair bulb melanocytes, as previously reported using a non-commercial antibody [20]. Here, using two anti-DCT commercial antibodies, we localized DCT protein expression (by IHC and WB) to melanocytes of the human proximal bulb region, including to melanocytes that appear to not yet be actively engaged in melanosome biogenesis, given the lack of expression of the melanosome antigen gp100. We further confirmed the expression of this key component of the enzymatic pathway for melanogenesis in ex vivo HF organ cultures and found that this protein could be upregulated in the proximal bulb region of human HFs after exposure to stress signal mediators, like pro-inflammatory IFNγ and UVB. This supports the previously reported view of DCT as a multi-functional protein, exerting functions beyond basal melanogenesis, including as a stem cell, and oxidative stress response modifier [42,43]. It additionally supports the view that stimulation of DCT levels (and activity) in melanocyte subpopulations of the human scalp may enhance local stress handling (e.g., oxidative stress/DNA damage/inflammation) to protect against melanocyte apoptosis, during canities-associated hair greying. By the same token, less mature gp100-negative but DCT-positive melanocytes in the hair bulb, as detected in the current study, may reflect an enzymatically inactive DCT subpopulation that may be more vulnerable to stress handling.

In summary, our study provides new evidence that within the human anagen hair bulb in vivo melanocyte-lineage cells exhibiting immature or mature melanocyte marker expression co-exist. This implies a functional diversity in this region of the human scalp HF, beyond our traditional view of melanocyte functionality expressed as the capacity to make melanosomes (evidenced by gp100 and or Melan-A expression) and subsequently produce melanin. What these immature bulbar melanocytes are doing in this part of the HF remains to be elucidated. Could they represent an in-transit migratory population destined to mature and synthesize melanin, or could they contribute in other ways to melanocyte biology, i.e., as non-melanogenic pigment cells?

The role of SOX10 in the life history of adult bulbar melanocytes is also intriguing, given that the expression of this transcription factor is required for melanocyte/melanoblast commitment in utero [44]. Is SOX10 expression dispensable for some melanocytes in the adult hair bulb? Our study shows in vitro a subpopulation of c-KIT-negative but MITF-positive melanocytes that lack SOX10 protein expression. Lastly, the expression of the multi-functional protein DCT (i.e., as a melanogenic enzyme, oxidative stress modulator, etc.) does appear to be an obligate feature of melanocytes in the human anagen hair bulb, regardless of their differentiation status. Much remains to be explored in the multi-FMU landscape of the human scalp HF, but it is our view that we now need to move beyond the mouse-centric view of HF melanocyte biology.

## 4. Materials and Methods

### 4.1. Human HF Collection, Organ Histo-Culture and Follicular Melanocyte Isolation

Individual anagen HFs were isolated from the human occipital scalp from routine elective hair restoration surgeries after informed patient consent, adhering to the declaration of Helsinki principles following ethical approval granted by the UCD ethics committee (#LS-19-44). Samples were used as indicated in Supplementary Table S1. HFs were microdissected from scalp pieces of approximately 1 cm^2^ under a stereomicroscope and cultured for 24 h at 37 °C with 5% CO_2_ in minimal William’s media E (Gibco; Waltham, MA, USA), #32557020), supplemented with 10 mg/mL insulin (Sigma-Aldrich; St. Louis, MO, USA), #I6634), 10 ng/mL hydrocortisone (Sigma-Aldrich, #H0888), and 1% penicillin/streptomycin (Gibco, #15070063). After quality control, growing HFs were allocated randomly to the different experimental groups. At 24 h after isolation, supplemented William’s E media was replaced and the cultured hair follicles were treated with 50 IU/mL of IFN-γ (Stemcell technology; Vancouver, BC, Canada), #78020.1) for 24 h at 37 °C, 5% CO_2_. Furthermore, 24 h after isolation, HFs were exposed to UVB (280–320 nm) irradiation (dosage 402 mJ/cm^2^), by using a commercial UVB bulb. Treated HFs were collected for OCT preservation and protein extraction. For follicular melanocyte isolation, individual hair follicles (at least 20 HFs) were treated according to methods described by Tobin et al. [13,14]. Briefly, after microdissection and treatment of the HF sample with collagenase (Gibco, #17104019) for 2 h at 37 °C, a cell suspension was obtained by 3 cycles of 0.05% trypsin/EDTA digestion (Gibco, 2530054). Single-cell suspensions were magnetically labeled with CD117 Human microbeads, according to the manufacturer’s instructions (Miltnyi Biotec; Somerville, MA, USA), #130091332). Both CD117^+^ and CD117^-^ fractions were collected, and cells were cultured in melanocyte media (2:1 media; MEM supplemented with 2% FBS, 1% non-essential amino acids, 5 ngml^−1^ of endothelin-1 and bFGF, 1% penicillin/streptomycin, and complete keratinocyte growth medium (Promocell, Heidelberg, Germany)) at 37 °C, 5% CO_2_.

### 4.2. Frozen Hair Sample Processing and Immunohistochemistry

Hair follicle samples embedded in an OCT matrix were processed for longitudinal sections of 8–10 µm, (Leica cryostat). After air-drying for 10 min, cryosections were fixed with acetone at −20 °C for 10 min and preincubated with blocking buffer (PBS + 5% donkey serum + 0.5% BSA) for one hour at room temperature prior to incubation with primary antibodies overnight at 4 °C (see Table S2). Secondary antibody incubation was performed for one hour at 37 °C with fluorescent-conjugated antibodies (see Table S2), and slides were mounted with antifade VectorShield mounting medium with DAPI (Vector laboratories; Newark, CA, USA), H-1500). Immunostaining visualization was performed using an IX83 Inverted Fluorescent Microscope (Olympus; Hamburg, Germany). Images were taken using Olympus cellSens3.2 (Build23706) Software and processed with ImageJ1.49s software. For DCT/TRP2 immunodetection, the fixation step was performed with 7% paraformaldehyde for 10 min.

### 4.3. Protein Extraction and Western-Blotting

To prepare HF protein extracts, the selected regions of HFs (mid–lower; Figure 1) were collected and lysed with 8M urea buffer (8M urea; tris-Hcl pH 7.5) supplemented with protease and phosphatase inhibitors (Roche: #11836170001; 04906837001) and kept for 4 h at 4 °C. Total protein extracts were obtained by using an ultrasonic homogenizer (Syclon SKL-150w; 2 cycles of 2 s on/off), followed by centrifugation at 10,000× *g* for 10 min at 4 °C. Protein concentration was determined using the DC protein Assay Kit (Bio-Rad, Hercules, CA, USA) and an equal amount of each sample (40 µg) was boiled at 95 °C for 5 min. Total proteins were separated on SDS-PAGE gels, transferred onto nitrocellulose membranes in transfer buffer (25 mM Tris; 192 mM glycine; 0.5% SDS; 20% Ethanol) for 120 min at 100 V, and processed for immunoblot analysis. Membranes were blocked with 5% skimmed milk in TBST (Tris-buffered saline containing 0.1% Tween-20) for 1 h at room temperature and incubated with primary antibodies overnight at 4 °C (Supplementary Table S1). After the washing steps, blots were incubated with the secondary antibodies HRP-conjugated secondary antibodies (Supplementary Table S1) for 1 h at room temperature. Supersignal West Pico/Femto chemiluminescence substrate (Thermo Fisher Scientific; Waltham, MA, USA) #34577; #34095) and G:Box XRQ gel doc system (SynGene, Bangalore, India) were used for image capturing. Band intensities were quantified using ImageJ software. Quantitative normalization was achieved using β-actin or GAPDH antibodies.

### 4.4. Statistical Analysis 

Numerical data are presented as mean ± standard deviation (SD). Statistical tests used for comparing different data sets are indicated. GraphPad Prism software (version 9.00; San Diego, CA, USA) was used to perform the statistical tests.

## Data Availability

Not applicable.

## Supplementary Materials

The following supporting information can be downloaded at: https://www.mdpi.com/article/10.3390/ijms241612809/s1.

## References

[1] Paus R. (1998). Principles of Hair Cycle Control. J. Dermatol..

[2] Slominski A., Wortsman J., Płonka P.M., Schallreuter K.U., Paus R., Tobin D.J. (2005). Hair Follicle Pigmentation. J. Investig. Dermatol..

[3] Christoph T., Müller-Röver S., Audring H., Tobin D.J., Hermes B., Cotsarelis G., Rückert R., Paus R. (2000). The Human Hair Follicle Immune System: Cellular Composition and Immune Privilege. Br. J. Dermatol..

[4] Tobin D.J. (2010). The Cell Biology of Human Hair Follicle Pigmentation. Pigment. Cell Melanoma Res..

[5] Slominski A., Paus R. (1993). Melanogenesis Is Coupled to Murine Anagen: Toward New Concepts for the Role of Melanocytes and the Regulation of Melanogenesis in Hair Growth. J. Investig. Dermatol..

[6] Steingrímsson E., Copeland N.G., Jenkins N.A. (2005). Melanocyte Stem Cell Maintenance and Hair Graying. Cell.

[7] Vandamme N., Berx G. (2019). From Neural Crest Cells to Melanocytes: Cellular Plasticity during Development and Beyond. Cell. Mol. Life Sci..

[8] Casalou C., Moreiras H., Mayatra J.M., Fabre A., Tobin D.J. (2022). Loss of “Epidermal Melanin Unit” Integrity in Human Skin During Melanoma-Genesis. Front. Oncol..

[9] O’Sullivan J.D.B., Nicu C., Picard M., Chéret J., Bedogni B., Tobin D.J., Paus R. (2020). The Biology of Human Hair Greying. Biol. Rev. Camb. Philos. Soc..

[10] Tobin D.J., Bystryn J.-C. (1996). Different Populations of Melanocytes Are Present in Hair Follicles and Epidermis. Pigment. Cell Res..

[11] Grichnik J.M., Ali W.N., Burch J.A., Byers J.D., Garcia C.A., Clark R.E., Shea C.R. (1996). Kit expression reveals a population of precursor melanocytes in human skin. J. Investig. Dermatol..

[12] Grichnik J.M., Burch J.A., Burchette J., Shea C.R. (1998). The SCF/KIT Pathway Plays a Critical Role in the Control of Normal Human Melanocyte Homeostasis. J. Investig. Dermatol..

[13] Tobin D.J., Colen S.R., Bystryn J.-C. (1995). Isolation and Long-Term Culture of Human Hair-Follicle Melanocytes. J. Investig. Dermatol..

[14] Kauser S., Thody A.J., Schallreuter K.U., Gummer C.L., Tobin D.J. (2004). B-Endorphin as a Regulator of Human Hair Follicle Melanocyte Biology. J. Investig. Dermatol..

[15] Grützkau A., Radbruch A. (2010). Small but Mighty: How the MACS^®^-Technology Based on Nanosized Superparamagnetic Particles Has Helped to Analyze the Immune System within the Last 20 Years. Cytom. Part A.

[16] Bashamboo A., Taylor A.H., Samuel K., Panthier J.-J., Whetton A.D., Forrester L.M. (2006). The Survival of Differentiating Embryonic Stem Cells Is Dependent on The SCF-KIT Pathway. J. Cell Sci..

[17] Lennartsson J., Rönnstrand L. (2012). Stem cell factor receptor/c-kit: From basic science to clinical implications. Physiol. Rev..

[18] Nishimura E.K., Jordan S.A., Oshima H., Yoshida H., Osawa M., Moriyama M., Jackson I.J., Barrandon Y., Miyachi Y., Nishikawa S.-I. (2002). Dominant role of the niche in melanocyte stem-cell fate determination. Nature.

[19] Sun Y., Song M., Stevanović S., Jankowiak C., Paschen A., Rammensee H.-G., Schadendorf D. (2000). Identification of a New HLA-A*0201-Restricted T-Cell Epitope from the Tyrosinase-Related Protein 2 (TRP2) Melanoma Antigen. Int. J. Cancer.

[20] Commo S., Gaillard O., Thibaut S., Bernard B.A. (2004). Absence of TRP-2 in Melanogenic Melanocytes of Human Hair. Pigment. Cell Res..

[21] Körner A.M., Pawelek J.M. (1980). Dopachrome conversion: A possible control point in melanin biosynthesis. J. Investig. Dermatol..

[22] Kawakami A., Fisher D.E. (2011). Key Discoveries in Melanocyte Development. J. Investig. Dermatol..

[23] Liao C.-P., Booker R.C., Morrison S.J., Le L.Q. (2017). Identification of Hair Shaft Progenitors That Create a Niche for Hair Pigmentation. Genes Dev..

[24] Nishimura E.K., Granter S.R., Fisher D.E. (2005). Mechanisms of Hair Graying: Incomplete Melanocyte Stem Cell Maintenance in the Niche. Science.

[25] Tobin D.J. (2009). Aging of the Hair Follicle Pigmentation System. Int. J. Trichology.

[26] Kauser S., Westgate G.E., Green M.R., Tobin D.J. (2011). Human Hair Follicle and Epidermal Melanocytes Exhibit Striking Differences in Their Aging Profile Which Involves Catalase. J. Investig. Dermatol..

[27] Sharov A., Tobin J.D., Sharova T.Y., Atoyan R., Botchkarev V.A. (2005). Changes in different melanocyte populations during hair follicle involution (catagen). J. Investig. Dermatol..

[28] Polisetti N., Schlötzer-Schrehardt U., Reinhard T., Schlunck G. (2020). Isolation and Enrichment of Melanocytes From Human Corneal Limbus Using CD117 (c-Kit) As Selection Marker. Sci. Rep..

[29] Li S., Zenkel M., Kruse F.E., Gießl A., Schlötzer-Schrehardt U. (2022). Identification, Isolation, and Characterization of Melanocyte Precursor Cells in the Human Limbal Stroma. Int. J. Mol. Sci..

[30] Yoshida H., Kunisada T., Kusakabe M., Nishikawa S., Nishikawa S.-I. (1996). Distinct stages of melanocyte differentiation revealed by analysis of nonuniform pigmentation patterns. Development.

[31] Botchkareva N.V., Khlgatian M., Jack Longley B., Botchkarev V.A., Gilchrest B.A. (2001). Scf/C-Kit Signaling is required for cyclic regeneration of the hair pigmentation Unit. FASEB J..

[32] Peters E.M.J., Tobin D.J., Botchkareva N., Maurer M., Paus R. (2002). Migration of melanoblasts into the developing murine hair follicle is accompanied by transient c-Kit expression. J. Histochem. Cytochem..

[33] Kelsh R.N. (2006). Sorting out Sox10 Functions in Neural Crest Development. BioEssays.

[34] Cronin J.C., Watkins-Chow D.E., Incao A., Hasskamp J.H., Schönewolf N., Aoude L.G., Hayward N.K., Bastian B.C., Dummer R., Loftus S.K. (2013). SOX10 Ablation Arrests Cell Cycle, Induces Senescence, and Suppresses Melanomagenesis. Cancer Res..

[35] Shakhova O., Zingg D., Schaefer S.M., Hari L., Civenni G., Blunschi J., Claudinot S., Okoniewski M., Beermann F., Mihic-Probst D. (2012). Sox10 Promotes the Formation and Maintenance of Giant Congenital Naevi and Melanoma. Nat. Cell Biol..

[36] Harris M.L., Baxter L.L., Loftus S.K., Pavan W.J. (2010). Sox Proteins in Melanocyte Development and Melanoma. Pigment. Cell Melanoma Res..

[37] Michalak-Mićka K., Büchler V.L., Zapiórkowska-Blumer N., Biedermann T., Klar A.S. (2022). Characterization of a Melanocyte Progenitor Population in Human Interfollicular Epidermis. Cell Rep..

[38] Kauser S., Thody A.J., Schallreuter K.U., Gummer C.L., Tobin D.J. (2005). A Fully Functional Proopiomelanocortin/Melanocortin-1 Receptor System Regulates the Differentiation of Human Scalp Hair Follicle Melanocytes. Endocrinology.

[39] Potterf S.B., Furumura M., Dunn K.J., Arnheiter H., Pavan W.J. (2000). Transcription Factor Hierarchy in Waardenburg Syndrome: Regulation of MITF Expression by SOX10 and PAX3. Hum. Genet..

[40] Ludwig A., Rehberg S., Wegner M. (2004). Melanocyte-Specific Expression of Dopachrome Tautomerase Is Dependent on Synergistic Gene Activation by the Sox10 and Mitf Transcription Factors. FEBS Lett..

[41] Guyonneau L., Murisier F., Rossier A., Moulin A., Beermann F. (2004). Melanocytes and Pigmentation Are Affected in Dopachrome Tautomerase Knockout Mice. Mol. Cell. Biol..

[42] Seiberg M. (2013). Age-Induced Hair Greying—The Multiple Effects of Oxidative Stress. Int. J. Cosmet. Sci..

[43] Michard Q., Commo S., Belaidi J.-P., Alleaume A.-M., Michelet J.-F., Daronnat E., Eilstein J., Duche D., Marrot L., Bernard B.A. (2008). TRP-2 Specifically Decreases WM35 Cell Sensitivity to Oxidative Stress. Free. Radic. Biol. Med..

[44] Dilshat R., Nhung Vu H., Steingrímsson E. (2021). Epigenetic regulation during melanocyte development and homeostasis. Exp. Dermatol..

