# Supplementary material for: Beyond the Epidermal-Melanin-Unit: The Human Scalp Anagen Hair Bulb Is Home to Multiple Melanocyte Subpopulations of Variable Melanogenic Capacity
Source: Int J Mol Sci. 2023 Aug 15;24(16):12809. doi: 10.3390/ijms241612809 (PMC10454394; doi:10.3390/ijms241612809)
Supplement: Supplementary file 1 [file ijms-24-12809-s001.zip › ijms-2508333-SI.pdf]

Supplementary Materials
Supplementary Figure S1

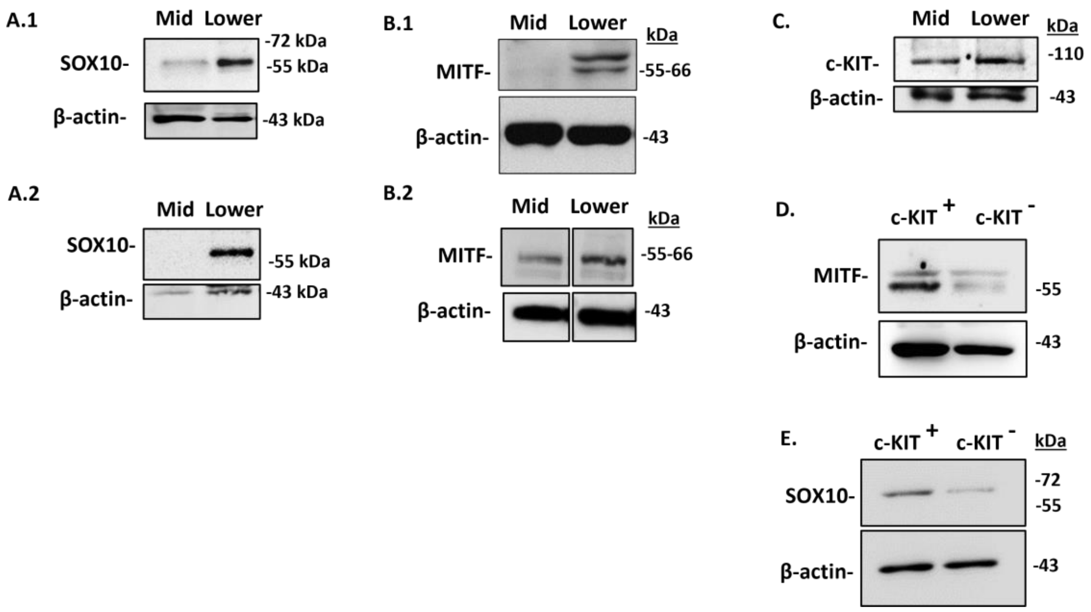

Figure S1. Expression of SOX10, MITF and c-KIT in the Mid and Lower regios of human HF. Replicas of the Western-blot analysis of Figure 3, using protein extracts from distinct HF donors (A-E).

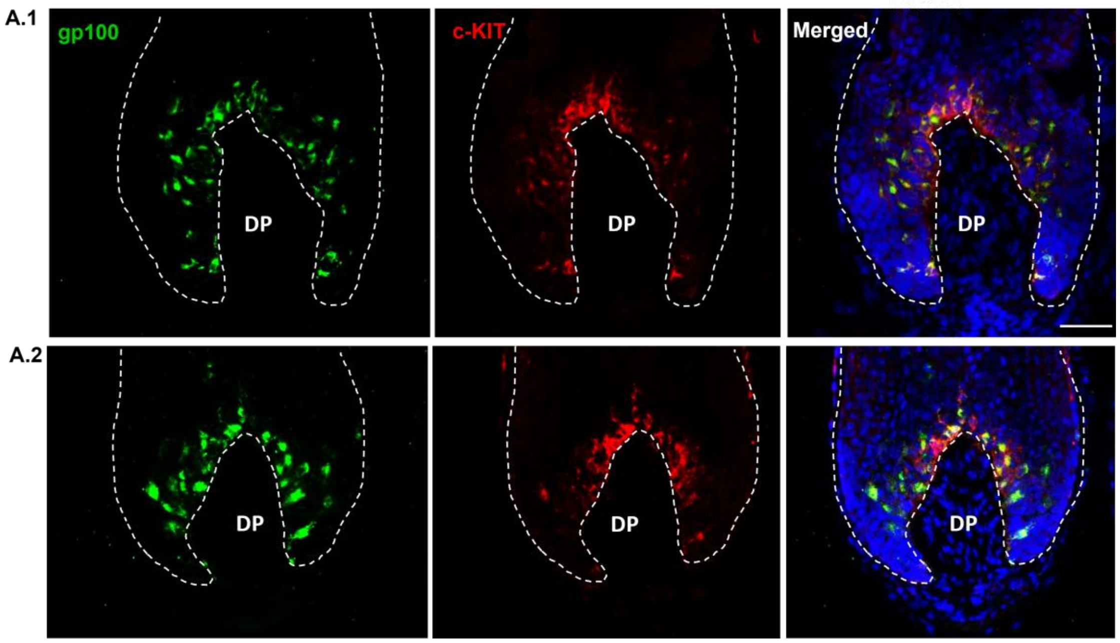

Figure S2. Expression of gp100 (green) and c-KIT (red) in human anagen VI bulb. Replicas images of Figure 4 (A.1 and A.2; n=2, distinct donors). Scale bar = 50 µm.

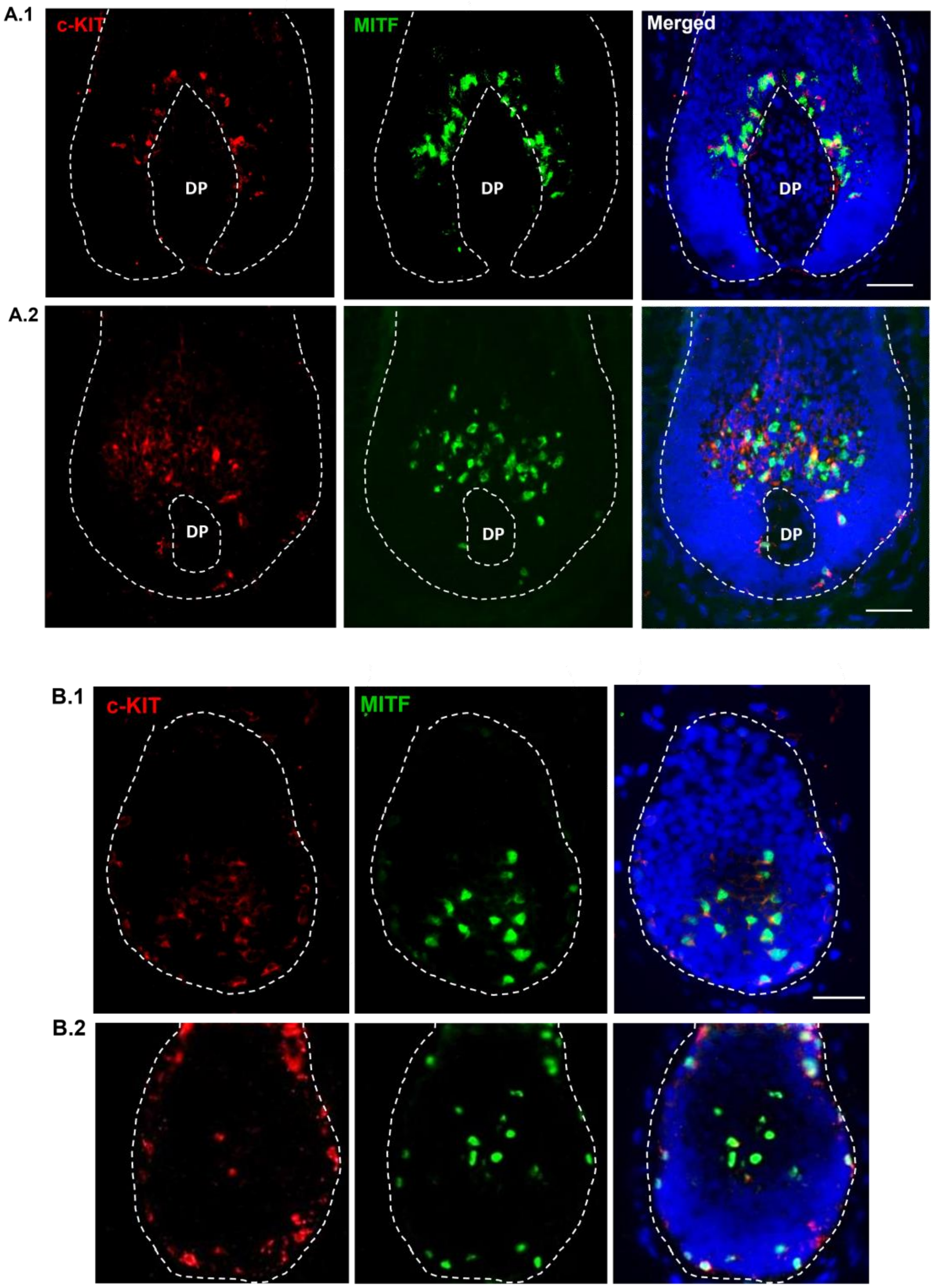

Figure S3. Expression of MITF (green) and c-KIT (red) in human anagen VI bulb.
Replicas of images of Figure 5 (A.1/B.1 and A.2/B.2; n=2, distinct donors). Scale bar = 50 μm.

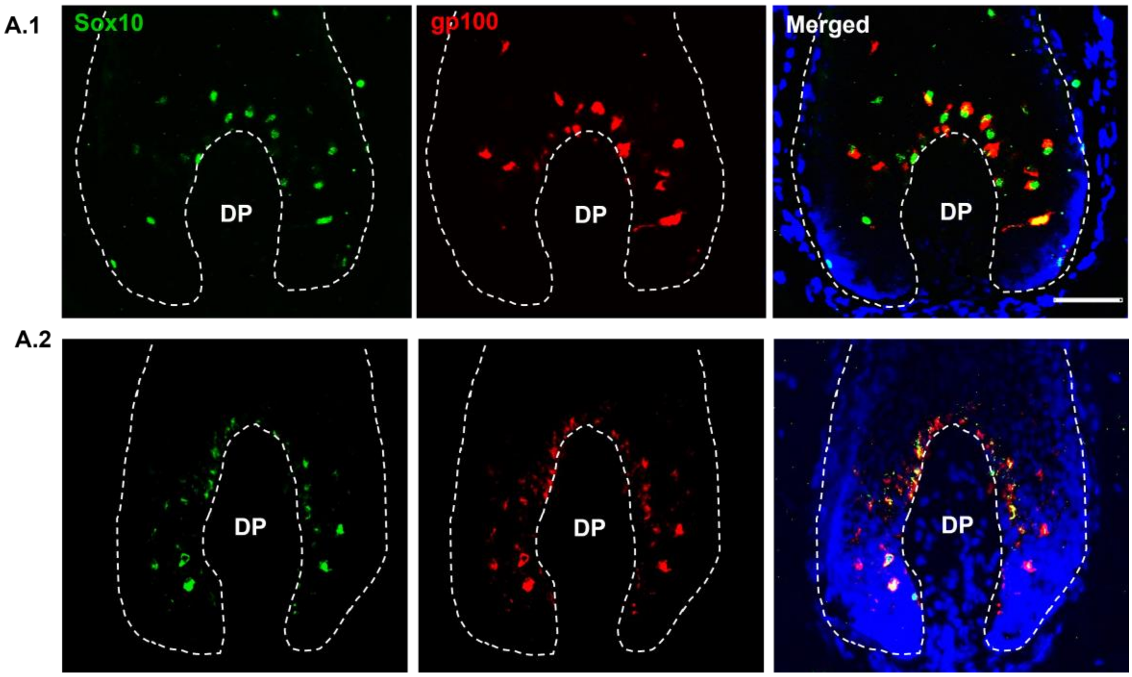

Figure S4. Expression of SOX10 (green) and gp100 (red) in human anagen VI bulb. Replicas of images of Figure 6A (A.1 and A.2; n=2, distinct donors). Scale bar = 50 µm.

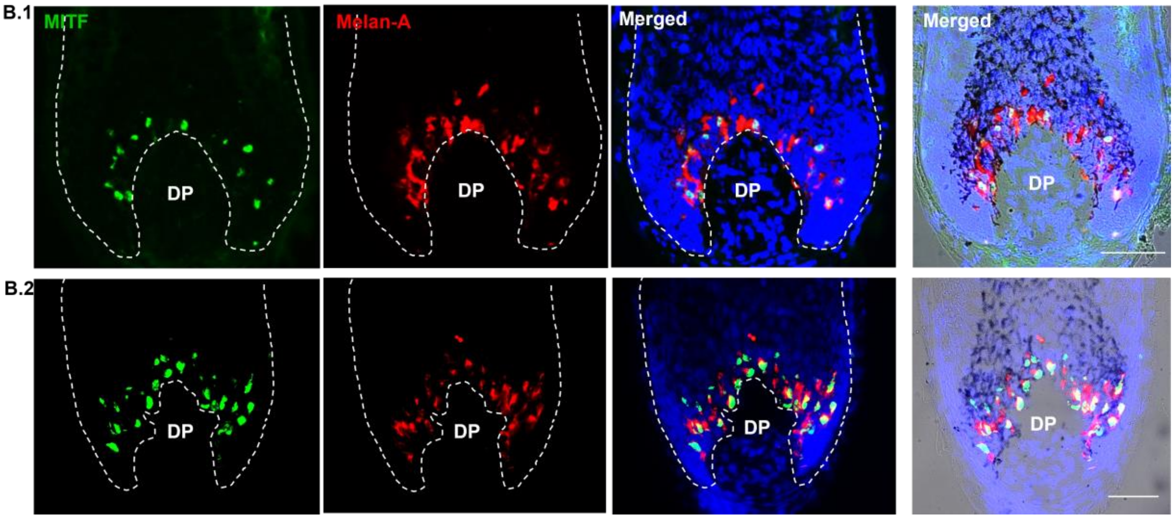

Figure S5. Expression of MITF (green) and MelanA (red) in human anagen VI bulb. Replicas of images of Figure 6B (B.1 and B.2; n=2, distinct donors). Scale bar = 50 µm.

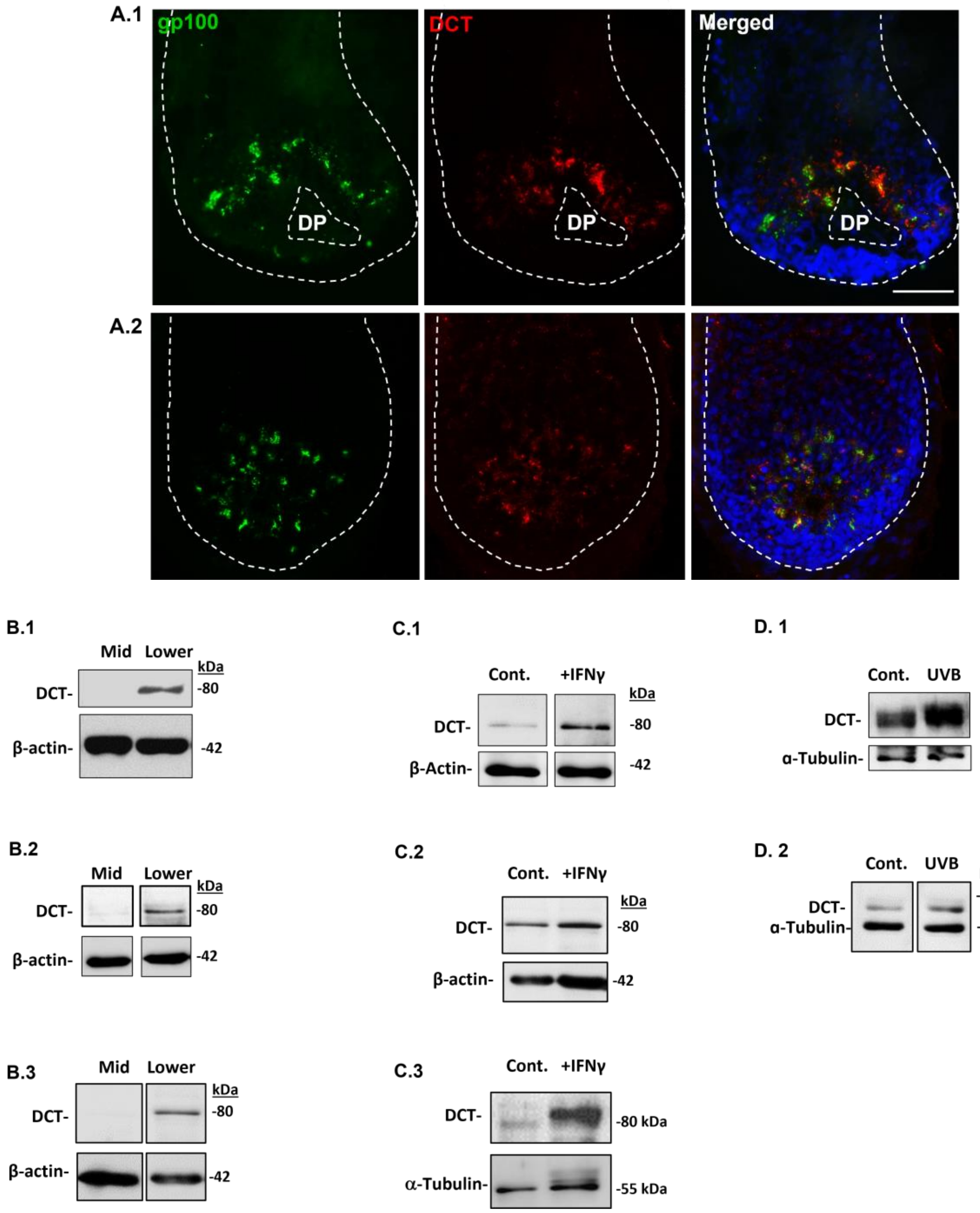

Figure S6. Expression of gp100 (green) and DCT (red) in human anagen VI bulb. (A) Replicas of images of Figure 7 (A.1 and A.2; n=2, distinct donors). Scale bar = 50 µm. (B) Expression of DCT in the Mid and Lower regions of human HF. Replicas of the Western-blot analysis of Figure 7, using protein extracts from distinct HF donors (n=3). (C,D) Expression of DCT upon HF stimuli/treatment with IFNγ and UVB radiation. N=3 for C1-3; n=2 for D1,2. β-actin and α-tubulin were used as loading controls.

Supplementary Table S1. Information of human hair follicles used for experiments (F—female; M—male; IHC- Immunohistochemistry; WB- Western-blot).

DONOR	AGE (YEARS)	SEX	HAIR COLOR	ETHNICITY	IHC	WB	CELL ISOLATION AND CULTURE
1	47	M	Brown to Black	Caucasian		x	
2	55	M	Brown to Black	Caucasian		x	
3	63	M	Brown to Black	Caucasian		x	
4	45	M	Brown to Black	Caucasian		x	
5	47	M	Brown to Black	Caucasian		x	
6	63	M	Brown to Black	Caucasian		x	
7	55	M	Brown to Black	Caucasian		x	
8	70	M	Brown to Black	Caucasian		x	
9	25	M	Brown to Black	Caucasian		x	
10	23	F	Brown to Black	Caucasian		x	
11	62	M	Brown to Black	Caucasian		x	
12	41	M	Brown to Black	Caucasian			x
13	44	M	Brown to Black	Caucasian			x
14	41	M	Brown to Black	Caucasian			x
15	53	M	Brown to Black	Caucasian	x		
16	38	M	Brown to Black	Caucasian	x		
17	27	F	Brown to Black	Caucasian	x		
18	26	M	Brown to Black	Caucasian	x		
19	45	M	Brown to Black	Caucasian	x		
20	37	M	Brown to Black	Caucasian	x		
21	47	M	Brown to Black	Caucasian	x		

Supplementary Table S2: Antibodies used in the study.

ANTIBODY	APPLICATION	ANTIBODY DILUTION	ANTIBODY SOURCE	IDENTIFIER
Gp100	IHC	1:25	Abcam	ab34165
c-KIT	IHC/WB	1:100 (IHC) 1:1000 (WB)	Abcam	ab32363
c-KIT	IHC	1:100	Bioorbit	Orb178436
SOX-10	IHC	1:50	R&D system	AF2864
SOX-10	WB	1:1000	Cell Signaling Tech.	69661
MITF	IHC	1:100	Cell marque	284M-94
MITF	WB	1:1000	Invitrogen	MA5-14154
TRP-2/DCT	IHC	1:100	Abcam	ab74073
TRP-2/DCT	WB	1:1000	Invitrogen	PA5-72571
α-ACTIN	WB	1:1000	Abcam	Ab8227
GAPDH	WB	1:1000	Invitrogen	MA5-15738
MELAN-A	IHC	1:1000	Abcam	ab210546
α-tubulin	WB	1:1000	Abcam	ab52866
HRP-rabbit	WB	1:4000	Cell Signaling Tech.	7074S
HRP-mouse	WB	1:4000	Cell Signaling Tech.	7076S
Chicken anti-Goat 488	IHC	1:200	Invitrogen	A-21467
Goat anti-Rabbit 555	IHC	1:200	Invitrogen	A32732
Donkey anti-Mouse 488	IHC	1:200	Invitrogen	A-21202
